# Correction: Alhoqail et al. Husk-like Zinc Oxide Nanoparticles Induce Apoptosis through ROS Generation in Epidermoid Carcinoma Cells: Effect of Incubation Period on Sol-Gel Synthesis and Anti-Cancerous Properties. *Biomedicines* 2023, *11*, 320

**DOI:** 10.3390/biomedicines14102163

**Published:** 2026-09-24

**Authors:** Wardah A. Alhoqail, Abdulaziz S. Alothaim, Mohd Suhail, Danish Iqbal, Mehnaz Kamal, Majid Mohammed Asmari, Azfar Jamal

**Affiliations:** 1 Department of Biology, College of Education, Majmaah University, Al-Majmaah 11952, Saudi Arabia; w.alhoqail@mu.edu.sa; 2 Department of Biology, College of Science in Zulfi, Majmaah University, Al-Majmaah 11952, Saudi Arabia; a.alothaim@mu.edu.sa; 3 King Fahd Medical Research Center, King Abdulaziz University, Jeddah 21589, Saudi Arabia; suhaildbt@gmail.com; 4 Department of Medical Laboratory Sciences, Faculty of Applied Medical Sciences, King Abdulaziz University, Jeddah 21589, Saudi Arabia; 5 Department of Health Information Management, College of Applied Medical Sciences, Buraydah Private Colleges, Buraydah 51418, Saudi Arabia; di.researchbio@gmail.com; 6 Department of Pharmaceutical Chemistry, College of Pharmacy, Prince Sattam Bin Abdulaziz University, Al-Kharj 11942, Saudi Arabia; mailtomehnaz@gmail.com; 7 Research Center, College of Pharmacy, King Saud University, Riyadh 11451, Saudi Arabia; masmary@ksu.edu.sa; 8 Health and Basic Science Research Centre, Majmaah University, Al-Majmaah 11952, Saudi Arabia

## Error in Figure

In the original publication [1], a concern was raised that the control image in Figure 8 overlapped with a related article [2], which described different experimental incubation times (12 h vs. 2–10 weeks). The authors have clarified that this overlap occurred unintentionally during the concurrent preparation of the two manuscripts.

To address this issue, the authors have repeated the experiments under identical conditions and provided new, original, uncropped images for Figure 8. The corrected Figure 8 appears below.

The authors state that the scientific conclusions are unaffected. This correction was approved by the Academic Editor. The original publication has also been updated.

## Figures and Tables

**Figure 8:**
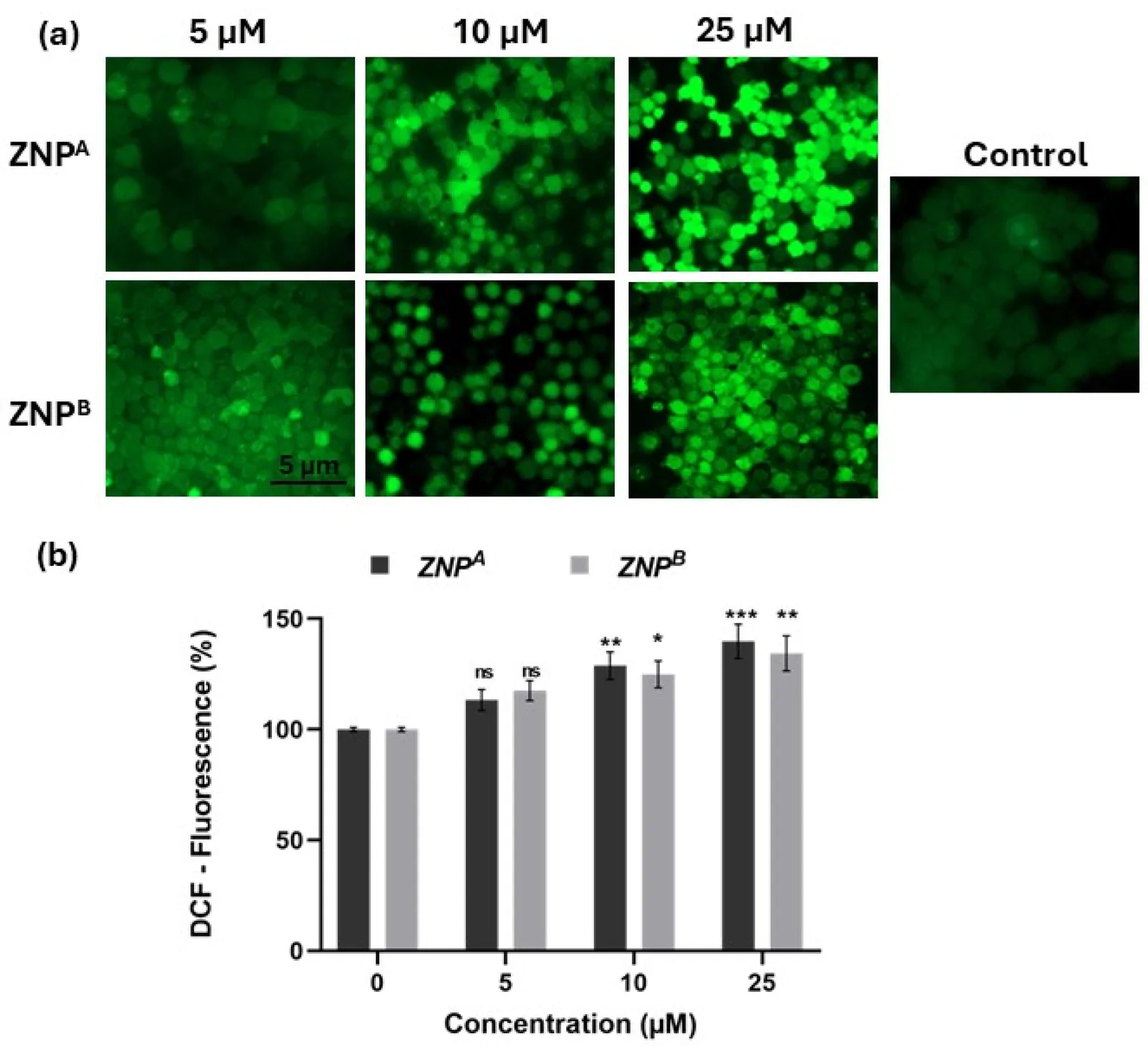
(**a**) Photomicrographs showing intracellular ROS generation in human epidermoid carcinoma A431 cell lines induced by control, 5 μM, 10 μM, and 25 μM concentrations of zinc oxide nanoparticles after 2 weeks for ZNP^A^ and 10 weeks for ZNP^B^, respectively, after 12 h incubation and staining with DCFH-DA. Scale bar = 50 μm. (**b**) Graph showing extent of ROS generation expressed as the percentage of fluorescence intensity relative to the control. Values are expressed as mean ± SEM of at least three independent experiments. * *p* < 0.01, ** *p* < 0.001, *** *p* < 0.0001, as compared with their respective control (ns, non-significant).
